# Articaine Versus Mepivacaine in Inferior Alveolar Nerve Block for Patients With Irreversible Pulpitis: A Systematic Review and Meta-Analysis of Randomized Controlled Trials

**DOI:** 10.7759/cureus.73360

**Published:** 2024-11-09

**Authors:** Meshari Alkandari, Mohammad Alshammari, Amnah Ghaleb, Talal Alshammari, Rawabi Alenezi, Shaikha Almutairi

**Affiliations:** 1 Dentistry, Rumaithiya Polyclinic, Ministry of Health, Kuwait City, KWT; 2 Dentistry, Ministry of Health, Jahra, KWT; 3 Dentistry, Saad Al-Abdullah Primary Health Care Center, Jahra Health Region Administration, Ministry of Health, Jahra, KWT; 4 Dentistry, Jahra Primary Health Care Center, Jahra Health Region Administration, Ministry of Health, Jahra, KWT

**Keywords:** articaine, ianb, irreversible pulpiti, mepivacaine, pain

## Abstract

Inferior alveolar nerve block (IANB) as an anesthetic strategy has shown conflicting results in terms of efficacy in the treatment of patients with irreversible pulpitis. Mepivacaine and articaine are anesthetic agents commonly used in the IANB technique for pulpal anesthesia. This review aimed to compare mepivacaine and articaine regarding pain and success rate. We conducted a search on the databases PubMed, Scopus, Web of Science (WOS), and Cochrane Central for randomized controlled trials (RCTs) assessing mepivacaine versus articaine until September 2024. The primary outcome of interest was success rate, while the secondary outcomes were pain intensity assessed by a 10-point visual analog scale (VAS) and incidence of severe pain. Data were pooled as odds ratio (OR) or mean difference (MD) with 95% confidence interval (CI) in a random-effect model using STATA.

Five RCTs including 568 patients were included in the final analysis. While there was no significant difference between the two studied groups regarding the success rate (OR: 0.92, 95% CI: 0.69 to 1.21, p=0.54), articaine significantly reduced the pain intensity compared to mepivacaine (MD: 0.59, 95% CI: 0.31 to 0.86], p<0.001). Moreover, no significant difference was observed regarding the incidence of severe pain. Articaine reduced the intensity of pain post-procedure, with comparable results regarding success rate and incidence of severe pain with mepivacaine. Further large-volume RCTs are warranted to study the differences between the two options in the long term.

## Introduction and background

Pulpitis is defined as an inflammation of the pulp, which is the soft inner tissue of the tooth that contains blood vessels, and nerves that supply the outer layers of the teeth [1]. Pulpitis is categorized into two types: reversible, where the tooth can be sealed with a filling; and irreversible, where the inflammation is severe and the tooth cannot be repaired [2]. Patients with irreversible pulpitis may experience severe pain due to acute pulp inflammation, increased vascular permeability, and vasodilation, thereby massively increasing the internal tissue pressure while being unable to expand [2]. Poor management could lead to anxiety and fear, subsequently postponing the treatment or complete avoidance [1].

Proper anesthesia during endodontic treatments is essential to ensure that the procedures are smooth and effective [3]. The inferior alveolar nerve block (IANB) is one of the most common anesthetic methods in the treatment of posterior mandibular teeth. In this technique, an anesthetic solution is injected into the pterygomandibular region to reach the inferior alveolar nerve and achieve pulp anesthesia. However, the rate of failure is substantial, estimated to range from 30 to 90% [4,5,6]. Several methods have been explored to improve the success rate of the IANB technique, such as the use of different local anesthetic agents, various administration techniques, premedication with analgesics, and supplementary approaches. One such supplementary technique, buccal infiltration (BI) injection, has proven effective in enhancing anesthesia success rates specifically for the mandibular molar region. However, a primary focus in many studies has been on using more potent anesthetic agents to achieve a higher success rate for IANB [7,8,9].

Different anesthetic agents such as ropivacaine, lidocaine, and articaine are widely used as local anesthetics during invasive surgeries owing to their high success rate and lower rates of complications [10-12]. Articaine has a thiophene ring instead of benzene, which increases its lipid solubility, thereby allowing enhanced entry through the nerve membrane and increasing the drug's efficacy [11,12]. On the other hand, mepivacaine has a faster onset and longer duration due to its lower ionization constant (pKa) than lidocaine. In this review, we aimed to provide a comprehensive assessment of the clinical difference between mepivacaine and articaine regarding clinical outcomes in patients with irreversible pulpitis who are undergoing the IANB technique.

## Review

Methodology

Study Design

This systematic review and meta-analysis was conducted according to the Preferred Reporting Items for Systematic Reviews and Meta-Analyses (PRISMA) [13] guidelines and the Cochrane Handbook [14]. Ethical approval is not required for this type of research.

Search Strategy and Data Sources

We systematically searched four databases - PubMed, Scopus, Web of Science (WOS), and Cochrane Central - from inception until September 2024 for all randomized controlled trials (RCTs) by using the following search strategy: ((“Mepivacaine”) AND (“Articaine”) AND (“IANB”) OR (“Inferior alveolar nerve block”) AND (“irreversible pulpitis”)). Detailed search terms for each database are illustrated in Table 1. Additionally, we performed a manual search based on the citations from ResearchGate, ClinicalTrials.gov, and the reference sections of the included studies for any relevant studies.

**Table 1:** Search terms used for each database

Database	Search terms	Search field	Search results
PubMed	((“Mepivacaine”) AND (“Articaine”) AND (“IANB”) OR (“Inferior alveolar nerve block”) AND (“irreversible pulpitis”))	All Field	215
Cochrane	((“Mepivacaine”) AND (“Articaine”) AND (“IANB”) OR (“Inferior alveolar nerve block”) AND (“irreversible pulpitis”))	All Field	355
Web of Science	((“Mepivacaine”) AND (“Articaine”) AND (“IANB”) OR (“Inferior alveolar nerve block”) AND (“irreversible pulpitis”))	All Field	219
SCOPUS	((“Mepivacaine”) AND (“Articaine”) AND (“IANB”) OR (“Inferior alveolar nerve block”) AND (“irreversible pulpitis”))	All Field	279

Eligibility Criteria and Endpoints

We included all RCTs meeting the following PICO criteria: 1) Patients: patients with irreversible pulpitis; 2) Intervention: mepivacaine as an intervention; 3) Comparison: articaine as a control; 4) Outcomes: the primary outcome of interest was the success rate. Moreover, secondary outcomes such as the assessment of postoperative pain evaluated by visual analog scale (VAS) and the incidence of severe pain were also assessed. The VAS tool is a 10-point scale with 0 indicating no pain at all and 10 indicating the worst pain ever [15]. We excluded single-arm studies, observational studies, narrative reviews, or published data from conference abstracts. Additionally, we excluded studies involving other local anesthetic agents such as lidocaine and ropivacaine.

Quality Assessment and Data Extraction

We used the Cochrane risk of bias assessment tool-2 (RoB-2) to evaluate the risk of bias in the included RCTs [16]. It includes five domains: randomization, deviations from indented interventions, missing data, measurement of outcomes, and reporting of results. Two reviewers independently evaluated the RoB-2 results and labeled the outcome as “high risk,” “low risk,” or “some concerns”. Any disagreements were resolved by consulting with another author.

We used an offline Excel sheet to extract data including baseline characteristics, a summary of included studies, and the studied clinical outcomes. The extracted baseline characteristics were as follows: sample size, age, male sex, and type of teeth, while the summary data included the location of the study, the methodology of drug administrations, and the studied outcomes.

Statistical Analysis

We pooled continuous data expressed as mean and standard deviation (SD) as mean difference (MD) with its 95% confidence interval (CI) in a random-effect model using the DerSimonian and Laird (DL) method, while the dichotomous data expressed as event and total were pooled as odds ratio (OR) with its 95% CI in a random-effect model using the DL method. A p-value less than 0.05 was considered statistically significant. We assessed the statistical heterogeneity by I-squared (I²) and p-value, of which I^2^ values ≥50% and p-values of less than 0.05 were considered significant heterogeneity. All statistical analyses were performed using the “meta esize” package in STATA 18MP. A publication bias test would be only implemented if 10 or more studies were included in the analysis where funnel plots would be visualized [17].

Results

Search Results

**Figure 1:**
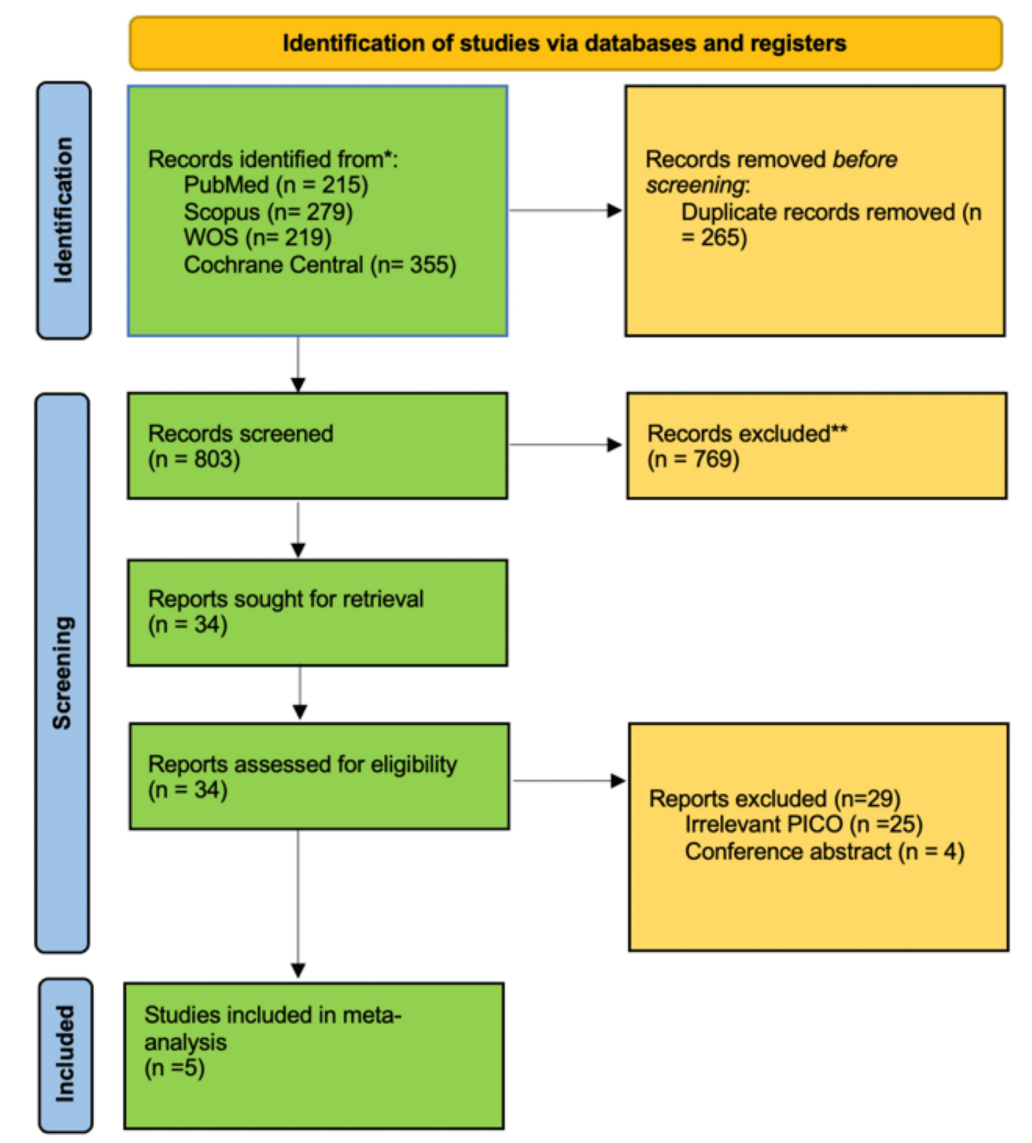
PRISMA flow diagram depicting the selection of studies PRISMA: Preferred Reporting Items for Systematic Reviews and Meta-Analyses

A total of 1,068 citations were retrieved from databases, of which 265 were removed as duplicates. After title and abstract screening, 34 citations were found eligible for full-text screening. Ultimately, five RCTs [18,19,20,21,22] were included in the final analysis. A PRISMA diagram depicting the selection of studies is shown in Figure 1.

Characteristics of the Included Studies and Risk of Bias

Five RCTs comprising 568 patients were included in the final analysis. Detailed data on administration protocol, type of teeth, and baseline characteristics of the included patients are summarized in Table 2 and Table 3.

**Table 2:** Summary of RCTs included in the study RCT: randomized controlled trial

Authors	Location	Study type	Intervention	Administration	Outcomes
Cunha et al., 2011 [18]	Brazil	RCT	Articaine- mepivacaine	4% articaine with 1:100,000 epinephrine OR 2% mepivacaine with 1:100,000 epinephrine	Success rate, incidence of severe pain
Allegretti et al., 2016 [19]	Brazil	RCT	Articaine- mepivacaine	4% articaine with 1:100,000 epinephrine OR 2% mepivacaine with 1:100,000 epinephrine	Pain score, success rate, incidence of severe pain
Habib et al., 2023 [20]	Egypt	RCT	Articaine- mepivacaine	2% mepivacaine hydrochloride with 1:100 000 adrenalin OR 4% articaine hydrochloride with 1:100 000 adrenaline	Pain score, success rate, incidence of severe pain
Gao and Meng 2020 [19]	China	RCT	Articaine- mepivacaine	Intraligamental injection of 0.9 mL of 4% articaine with 1:100,000 adrenaline OR intraligamental injection of 1.8 mL of 2% mepivacaine with 1:100,000 adrenaline	Pain score, success rate, incidence of severe pain
Singhal et al., 2022 [20]	India	RCT	Articaine- mepivacaine	Buccal infiltration with 4% articaine with 1:100,000 epinephrine OR 4-site intraligamental injection with 4% articaine with 1:100,000 epinephrine OR buccal infiltration with 2% mepivacaine with 1:100,000 epinephrine OR 4-site intraligamental with 2% mepivacaine with 1:100,000 epinephrine	Success rate, incidence of severe pain

**Table 3:** Baseline characteristics of the included patients ^*^Mean ± SD NR: not reported; SD: standard deviation

Authors	Sample size, n		Age, years		Male gender, n (%)		Type of teeth, n		
	Mepivacaine	Articaine	Articaine	Mepivacaine	Articaine	Mepivacaine	First molar	Second molar	Third molar
Cunha et al., 2011 [18]	15	15	19-57		19 (31%)		NR		
Allegretti et al., 2016 [19]	22	22	28.7 ± 8.09^*^	33.9 ± 9.49^*^	12 (54.5%)	6 (27.2%)	34	32	NR
Habib et al., 2023 [20]	165	165	29.89 ± 8.66^*^	29.20 ± 8.75^*^	42 (25.5%)	56 (33.9%)	203	123	4
Gao and Meng, 2020 [19]	52	52	39. 2 ± 13.2^*^	39.6 ± 13.0^*^	24 (46.2%)	22 (42.3%)	73	55	NR
Singhal et al., 2022 [21]	30	30	18-50		NR		NR		

Risk of Bias in the Included RCTs

**Figure 2:**
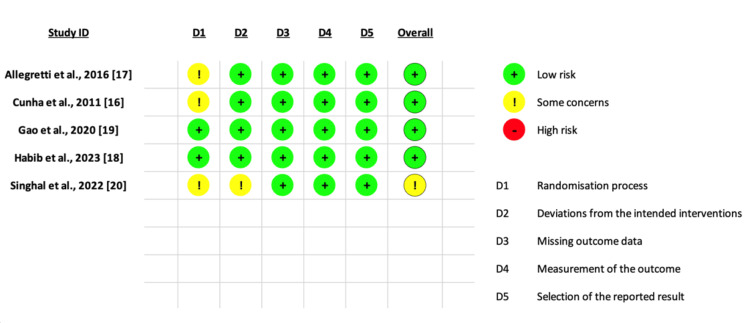
Risk of bias (RoB) graph relating to RCTs included in the study RCT: randomized controlled trial

Four RCTs were of low risk of bias and only one RCT showed some concerns. A detailed summary of RoB2 is shown in Figure 2.

Outcomes

**Figure 3:**
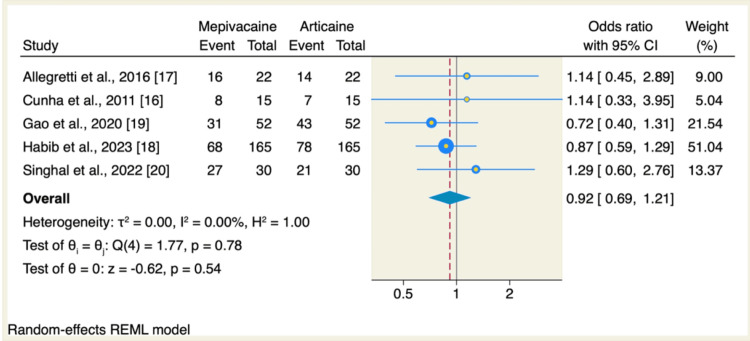
Meta-analysis comparing the success rate (%) of the mepivacaine and articaine groups CI: confidence interval

All five RCTs assessed the success rate; it was 52.82% (150 of 284 patients) in the mepivacaine group and 57.39% (163 of 284 patients) in the articaine group. There was no significant difference between mepivacaine and articaine regarding the success rate (OR: 0.92, 95% CI: 0.69 to 1.21, p=0.54; I^2^=0.00, p=0.78), as shown in Figure 3. The pooled analysis was homogenous.

**Figure 4:**
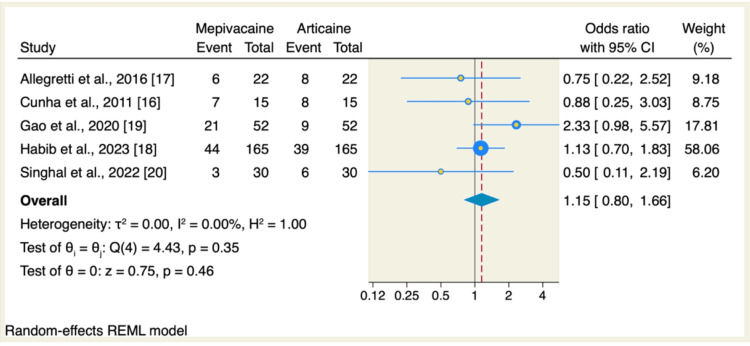
Meta-analysis comparing the incidence of severe pain (%) between the mepivacaine and articaine groups CI: confidence interval

Also, no significant difference in terms of the incidence of severe pain was reported between mepivacaine and articaine groups (OR: 1.15, 95% CI: 0.8 to 1.66, p=0.46; I2=0.00, p=0.35), as shown in Figure 4. The pooled analysis was homogenous. 

**Figure 5:**
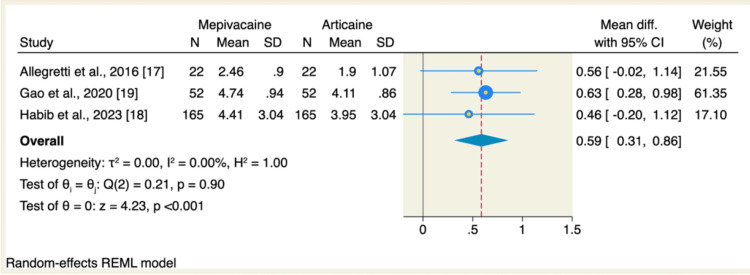
Meta-analysis comparing the intensity of pain between the mepivacaine and articaine groups as evaluated by 10-point VAS CI: confidence interval; SD: standard deviation; VAS: visual analog scale

On the other hand, articaine significantly reduced the intensity of postoperative pain, as evaluated by VAS, compared to mepivacaine (MD: 0.59, 95% CI: 0.31 to 0.86, p<0.001; I^2^=0.00, p = 0.9), as shown in Figure 5. The pooled analysis was homogenous.

Discussion

This meta-analysis is the first and the most comprehensive study assessing the difference between mepivacaine and articaine in patients with irreversible pulpitis; it included five RCTs comprising 568 patients. Our pooled estimate showed that there was no significant difference between mepivacaine and articaine regarding the primary outcome, the success rate, and the incidence of severe pain; however, articaine reduced the intensity of pain, as assessed by VAS, compared to mepivacaine, highlighting its superiority during IANB in patients with irreversible pulpitis.

Ensuring sufficient local anesthesia during the endoscopic procedures is important for reducing pain sensation, thereby reducing patients’ anxiety and also ensuring patients’ satisfaction [23,24]. Although IANB is a widely used local anesthesia in the mandible, it is associated with a high failure rate, which could be attributed to the anatomical variations of the mandibular foramen and innervations. On the other hand, BI has emerged as an alternative approach to IANB; however, it cannot yet replace the application of IANB as evidenced by a critical appraisal by Bartlett and Mansoor [24]. Yet, the literature has sufficient data on mepivacaine BI compared to articaine BI in patients with irreversible pulpitis.

In our study, the overall success rate was comparable between the two interventions without notable differences. Gao et al. [21] reported that supplementary articaine BI was associated with a higher success rate compared to mepivacaine BI after IANB injection. This could be explained by the fact that articaine contains a thiophene ring instead of the regular ring of benzene found in lidocaine; the thiophene ring might be associated with a higher success rate or articaine [25,26,27] as it allows for a greater lipid solubility, which in turns reflects the high amount of dose that can enter the neurons after articaine administration [12]. Another study by Habib et al. [20] reported that the success rate was 35.6% and 41.2% for mepivacaine and articaine, respectively. The success rate of mepivacaine in the literature ranges from 53.3% to 68.2% [18,28] while that of articaine ranges from 46.7% to 77.5% [18,19,29,30]. The difference between Habib et al's study and other studies could be attributed to the difference in the preoperative pain assessment where most of their patients (81.2%) reported severe pain while the rest reported moderate pain as assessed by VAS, and also the diagnostic criteria, where these factors play a significant role in terms of success rate [31].

Although there are similarities between mepivacaine and articaine regarding success rate, pain intensity was reduced in the articaine arm vs. the mepivacaine arm. This difference could be due to their different properties as to pharmacokinetics and their mechanism of action, which could guide the clinical decision [32,33]. Mepivacaine's shorter duration of soft tissue anesthesia makes it useful in pediatric dentistry, where children may chew their lips post-procedure [34]. Articaine, rapidly absorbed and inactivated via hydrolysis, has the shortest metabolic half-life among dental anesthetics (~27-42 minutes) [34]. Moreover, mepivacaine has a 60-90 min block anesthesia duration, while that of articaine is 90-120 min [33,34]. Also, articaine, with its unique chemical form including the thiophene ring, has higher anesthetic potency [32]. Our findings align with Gao et al. [21] who reported that articaine showed lower VAS ratings vs. mepivacaine in a post-hoc analysis. Their findings were explained by the unique characteristics of the articaine intervention. Moreover, they used intraligamentary injection to perform the BIs (there are two locations of injection: intraligamentary and subperiosteal). A study by Subramaniam et al. [35] found no significant difference between intraligamentary and subperiosteal routes in controlling tooth pain, indicating no difference in VAS ratings.

The severity of preoperative pain is one of the common indicators of anesthesia failure. According to a study by Aggarwal et al. [4], preoperative pain is proportional to the higher rate of IANB anesthetic failure, as patients with severe preoperative pain are likely to experience anesthetic failure at a higher rate compared to patients with mild pain. Moreover, the presence of preoperative pain was associated with higher VAS ratings within 24 hours before and after treatment [36,37]. To better understand the IANB anesthetic failure in patients with irreversible pulpitis, more large-volume RCTs that correlate the performance of mepivacaine with the neurophysiology of the pulp should be performed [2].

Our study has a few limitations, which should be addressed in future studies. Primarily, even though we conducted the most comprehensive analysis of the subject to date, all the included studies featured small sample sizes. Hence, we recommend that future studies be done on a larger scale to validate the current findings. Secondly, the quantity of solutions was not analyzed separately due to insufficient data, which should also be addressed by future research by evaluating multiple arms of different solutions to obtain the optimal dosage. Finally, the degree of preoperative pain was not comparable between included studies, which could lead to skewed observations, and a separate analysis of the meta-regression on the preoperative pain status could not be assessed due to the limited number of included studies.

## Conclusions

Based on our findings, articaine provides superior analgesia compared to mepivacaine by reducing postoperative pain intensity, as indicated by VAS scores; however, both interventions had comparable efficacy in procedural success and severe pain incidence during endoscopic approaches. These findings highlight articaine as a reliable option for enhanced patient comfort. Further high-quality RCTs with standardized protocols are needed to confirm these results and guide anesthetic selection in endoscopic procedures.
